# Expansion of hematopoietic stem cells for transplantation: current perspectives

**DOI:** 10.1186/2162-3619-1-12

**Published:** 2012-05-14

**Authors:** Jessica A Schuster, Maria R Stupnikov, Gina Ma, Wenbin Liao, Raymond Lai, Yupo Ma, Jerell R Aguila

**Affiliations:** 1 Department of Pathology, The State University of New York at Stony Brook, Stony Brook, NY, 11794, USA; 2 Department of Laboratory Medicine and Pathology, University of Alberta and Cross Cancer Institute, Edmonton, AB, T6G 1Z2, Canada

**Keywords:** Stem cell expansion, Gene therapy, Epigenetics, Umbilical cord blood, HOXB4, SALL4, Notch signaling

## Abstract

Hematopoietic stem cells (HSCs) are rare cells that have the unique ability to self-renew and differentiate into cells of all hematopoietic lineages. The expansion of HSCs has remained an important goal to develop advanced cell therapies for bone marrow transplantation and many blood disorders. Over the last several decades, there have been numerous attempts to expand HSCs in vitro using purified growth factors that are known to regulate HSCs. However, these attempts have been met with limited success for clinical applications. New developments in the HSC expansion field coupled with gene therapy and stem cell transplant should encourage progression in attractive treatment options for many disorders including hematologic conditions, immunodeficiencies, and genetic disorders.

## Introduction

Hematopoietic stem cells (HSCs) are rare stem cells that have the ability to differentiate into specialized blood cells, including lymphocytes, red blood cells, and platelets [1]. While studies to successfully expand these cells have spanned over the last three decades, a routine method for ex vivo expansion of human HSCs is still not available. However, stem cell biology has progressed in recent years and thus ex vivo expansion of human HSCs should become possible in the near future.

Stem cell transplants are a type of stem cell therapy used to treat cancers such as lymphoma and leukemia and other blood-related diseases [2]. Currently, there have been various difficulties associated with ex vivo expansion methods to generate stem cells for clinical therapies. The principle goal of all ex vivo attempts to expand HSCs is to produce a qualitative and quantitative acceptable cell population for bone marrow transplantation. Earliest attempts at ex vivo expansion centered on the use of cytokines known to support lineage committed cells [3]. Many researchers believe that these factors also played a key role in HSC proliferation. Thus, researchers hypothesized that the addition or removal of specific cytokines was able to regulate the survival and expansion of the stem cell population [2,3]. In more recent studies, researchers have focused on factors critical for embryologic development and the importance of signaling pathways that influence HSC self-renewal and expansion [2]. In the near future, it may be demonstrated that the most comprehensive approach may be a combination of various optimized conditions. Ex vivo expansion of HSCs is crucial for their use in the treatment of malignant and non-malignant diseases. Today, HSC gene therapy continues to be a highly attractive treatment option for many disorders including hematologic conditions, immunodeficiencies, and genetic disorders [2].

## Umbilical cord blood vs. Peripheral blood stem cells

In most bone marrow transplant centers, peripheral blood stem cells (PBSCs) remain the preferred source of stem cells for many types of allogeneic transplants, in which matched related or unrelated donors are available [3,4]. In the last decade, HSCs derived from umbilical cord blood (UCB) have become more readily utilized for allogeneic transplantation. Due to the low number of recovered stem cells from UCB, the initial uses were directed toward pediatric therapies [4]. With the assistance of new technologies, UCB is now used as a new source of stem cells for adult patients in numerous transplant centers for the treatment of malignancies such as leukemia, bone marrow failure states such as myelodysplastic syndrome, metabolic storage diseases, and hemoglobinopathies [5]. This alternative source of HSCs may provide a solution for patients who require a potentially curative allogeneic transplant but lack a suitable adult donor [5].

The use of UCB is now standard practice in pediatrics and, in fact, some pediatric hematologists are now advocating the preferential use of UCB over peripheral blood stem cells [6]. By utilizing multiple cord blood transplants, UCB transplants have become an expanding practice in adults for the treatment of malignant diseases [6-8]. The advantages of UCB transplantation include rapid availability, low risk of infection transmission, absence of donor risk, and preservation of graft versus-malignancy effects despite the relatively lower risk of graft-versus-host disease (GVHD) because of more tolerance to HLA-mismatch [6,8]. The disadvantages of UCB transplantation are the limited cell dose and resultant delayed engraftment, as well as the lack of additional immune cells if donor lymphocytes are needed [8]. In addition, viral reactivation, with viruses such as CMV and HHV6, has been noted to be problematic with these transplants [7]. Finally, the nature of immune reconstitution following UCB transplants is yet another concern that has been raised [8]. Recent advances have improved results with UCB transplantation and outcomes with matched unrelated transplants.

Advancements in the field of UCB transplantation have moved attention from investigating its safety and feasibility to addressing a myriad of specific issues. New work in UCB transplantation has focused on extending access, ensuring quality, accelerating engraftment, and reviewing outcomes in specific subgroups of patients [8]. The major advance in extending this treatment modality to adults has been the use of double cord blood transplants [8]. Supported by a rapidly growing body of evidence demonstrating the safety and efficacy of UCB transplantation, this HSC source is now being used more frequently for the treatment of adult patients with hematologic malignancies [9]. After an initially limited utilization among adults in contrast to routine utilization in children who lack a suitable sibling donor, UCB transplantation for older and larger patients is steadily increasing and finding acceptance among adult transplant physicians [9,10].

UCB is increasingly being used as an alternative source of hematopoietic stem/progenitor cells (HSCs/HPCs) for allogeneic bone marrow transplantation [9,10]. This is especially important for minority patients and patients of mixed ethnicity. However, delayed engraftment remains a significant problem, largely due to relatively low nucleated cell doses available in single UCB units, particularly for adults. Only a minority of adult patients has a UCB unit with a "satisfactory" cell dose to meet the requirement for allogeneic bone marrow transplantation [10,11]. A number of strategies are being investigated to overcome the cell-dose barrier, including ex-vivo expansion of UCB stem cells, intraosseous infusion of UCB cells, activating homing receptors (such as CXCR4), and use of double cord blood units [9-11]. To date, attempts at ex vivo expansion of hematopoietic stem cells have been met with only limited success. To overcome the cell–dose barrier, the combination of two UCB units is becoming commonplace in adolescent and adult populations [11]. The use of multiple UCB has yielded many more UCB transplants to treat adults, and this should be considered an important advance in the field of UCB transplantation [9,10]. However, when two or more UCBs are transplanted into one recipient, it is common for only one to be engrafted into the patient [9-11]. The engrafting UCB unit cannot be predetermined and molecular mechanisms surrounding this finding are still unclear. In some studies, the use of two UCB units appears to have a positive impact on outcomes; however, delayed engraftment continues to pose a significant risk for patients and increased GVHD is also noted [9-11] . An additional disadvantage of using multiple units is substantial increases in costs compared to the use of one UCB unit[11]. A possible way to improve outcome and extend applicability of UCB transplantation is via ex vivo or in vivo expansion and enhancement of homing.

## Ex vivo expansion of HSCs

In a publication in 2010, researchers described requirements which needed to be fulfilled when considering in vitro expansion of HSCs: (1) HSCs must be able to expand on a larger scale without sacrificing their self-renewal ability; (2) Expansion of HSCs must be safe and transplantable, and requires the method to be free of feeder cells, serum proteins, or microbial agents [12]. In order to determine optimal conditions for in vitro expansion of HSCs, investigators have adjusted various parameters in the hopes of increasing the number of engraftable stem cells. It is known that stem cells in the bone marrow are found in niches created by non-stem cells, and that these stem cells will remain undifferentiated and appear immortal as long as they do not leave the niche [5]. Therefore, the signaling pathways occurring in this niche are important to understand and examine. The manipulation of these signaling pathways for genes such as Notch, HOX-B4, and Wnt [12,13] have shown some positive results for ex vivo expansion. Others have shown that overexpression of genes, such as SALL4 [1] have the capacity to substantially increase the number of HSCs/HPCs in vitro. A TAT-SALL4 fusion protein (SALL4 protein fused with the TAT cell-penetrating peptide) has also been shown to rapidly expand HSCs/HPCs, making it feasible to translate this finding into the clinical setting [1]. Other experimental trials have tried to expand HSCs/HPCs with aryl hydrocarbon receptors, chelators, stromal coculture, and automated, continuous perfusion culture systems or "bioreactors" [1].

Notch proteins are important for the survival, self-renewal, and lineage determination of stem cells [12]. There are four Notch receptors (Notch1, Notch2, Notch3, and Notch4), five ligands (Jagged-1, Jagged-2, Delta-like-1, Delta-like-2, Delta-like-3, and Delta-like-4), and several modifier proteins [13] that constitute the Notch signaling network in vertebrates. A role for Notch in hematopoiesis was initially described by detection of the human Notch1 gene in CD34+ hematopoietic cells [14]. It has been demonstrated that manipulating the signaling pathway for the Notch gene plays a role in HSC/HPC growth and expansion. Several studies have found that this signaling network can augment HSCs/HPCs in vitro and lead to a 100-fold increase in CD34+ precursors [12]. Numerous preclinical murine studies involving Notch have been translated into human studies utilizing an engineered Notch ligand for ex vivo generation of CD34+ cells [14]. In fact, the engineered Notch ligand approach for ex vivo expansion is now under clinical investigation. In this phase 1 clinical trial, patients undergoing a myeloablative double cord blood transplantation are receiving one non-manipulated cord blood unit along with a second cord blood unit that has undergone Notch-mediated ex vivo expansion [15]. Early results show that patients may have better outcomes who undergo these mixed-unit treatments; however, long-term engraftment was still not achieved.

The homeobox gene family member HOXB4 has been shown to be a regulator of hematopoietic differentiation [16]. It is currently the most investigated transcription factor for its potential to increase the self-renewal properties of HSCs. The HOXB4 gene has been demonstrated to stimulate expansion of HSCs both in vivo and in vitro while still allowing HSCs to differentiate for both normal and myeloid cells [13]. In addition, overexpression through cellular modification of HOXB4 has led to expansion of both murine and human hematopoietic stem and progenitor cells. Human UCB CD34+ cells treated with HOXB4 fusion proteins have resulted in a 2.5-fold increase in long-term repopulating cells compared to uncultured controls [16]. Utilizing a more stable form of this protein may prove to be one effective strategy for ex vivo expansion in the future [16].

Wnt proteins are secreted morphogens that are essential for basic developmental processes, such as progenitor-cell proliferation, cell-fate specification, and the control of asymmetric cell division in various tissues [17]. Wnt has been found to stimulate in vitro expansion of HSCs/HPCs [18]. Prostaglandin E_2_ (PGE_2_) is a multifunctional eicosanoid that has been shown to enhance the engrafting capabilities of HSCs [19] by increasing both the homing and self-renewal events of HSCs [20].

The transcription factor SALL4 is a member of the SALL gene family and has been reported to play an essential role in maintaining ES cell pluripotency through interaction with Oct4 and Nanog [21,22] and establishes a connection between ESCs and the self-renewal properties of HSCs [23,24]. Researchers have discovered that overexpression of SALL4 can expand ex vivo human mobilized HSCs from peripheral blood [1]. SALL4-transduced cells were capable of ex vivo expansion of both, CD34 + CD38- and CD34 + CD38+ cells and showed enhanced stem cell engraftment and long term repopulation capacity in NOD-SCID mice [1]. These findings may suggest a possible new venue for investigating mechanisms of stem cell self-renewal and achieving clinically significant expansion of human HSCs. However, further studies need to be performed in nonhuman primates and human clinical studies to further test the efficacy and safety of these methods, and to validate if these expanded cells are capable of reconstituting hematopoiesis in transplanted patients.

## Current problems with ex vivo expansion methods

Currently, there are various methods being utilized to expand HSCs ex vivo. These include the use of cytokine cocktails, copper chelators, exposure to signaling molecules, stromal support and overexpression of transcription factors [23]. To date, most advances utilizing these various techniques are restricted to murine models. While studies using human CD34+ cells transplanted into NOD/SCID mice have shown some positive engraftment results [25], a large animal model must still be tested to prove true long-term multilineage engraftment. Many researchers believe that transducing and expanding HSCs by using lentiviral vectors is the most promising tool for HSC-based gene therapy [25]. Yet these studies must clearly assess the risks of insertional mutagenesis before larger clinical trials can start. Furthermore, the amount of time HSCs are expanded in culture is an additional problem surrounding ex vivo expansion. Increasing the period of culture promotes increased cell division, which can also increase the likelihood of spontaneous mutations or cytogenetic abnormalities [25]. While success for transduced and expanded HSCs has been demonstrated in NOD/SCID mice, it will be extremely important for these studies to be successfully translated into large animal or patient studies that address safety and efficacy issues. Future focus should be placed on the development of a standardized clinical protocol to expand engraftable long-term HSCs [26].

## Gene therapy and HSCs

Since 1999, there has been an average of about 100 new gene therapy clinical trials annually pursuing treatments for infectious diseases, cancer, and genetic diseases [27,28]. Over this short period of time, new advancements have occurred utilizing HSC expansion for gene therapy. HSC gene therapy continues to be an attractive therapeutic option for hematologic conditions, immune diseases, and numerous genetic diseases. In addition, HSCs may conceivably be the preeminent entry point for gene therapy of hematopoietic and immune systems because genetically modified HSCs are long-lived cells that have the capability to transfer their therapeutic characteristics to their daughter cells [13,28]. While medical institutions worldwide are working on gene therapy via HSCs, there are several formidable roadblocks to successful outcomes.

Over the last decade, there have been positive results obtained in gene therapy using HSCs in murine models, but these achievements have not been able to be translated into successful outcomes in non-human primates and human patients [27,28]. One possibility is that there are fundamental differing mechanisms controlling hematopoiesis in small short-lived and larger long-lived animals [27,28]. Furthermore, the low efficiency of viral transduction of HSCs in human trials may severely decrease the level of chimerism or long-term repopulating ability of the cells [27].

HSCs from sources such as umbilical cord blood or peripheral blood are rather limited. Transplant physicians and researchers have recently shown much higher interest in the usage of UCB cells for transplants due to the advantages they hold over other sources. These include a non-invasive collection method, a high repopulating efficiency, and a less stringent HLA-matching requirement [28]. New studies have proposed that the best alternative for successful transplants may be to combine ex vivo expansion methods with genetic manipulations of blood cells. Investigators believe that a significant increase in safety and engraftment could be achieved if genetically modified HSCs were expanded while simultaneously screened for integration sites prior to transplantation [28]. These gene modified HSCs could be expanded with cytokines, signaling molecule exposure, copper chelators, or overexpression of transcription factors [23,28]. The most likely successful approach may be one which involves a combination of methods.

Despite new advances in HSC gene therapy, the lack of a proven gene-modified HSC drug or protocol emphasizes that many obstacles remain inexplicable [28]. The greatest challenge for in vivo studies may be successfully achieving a safe level of gene dosage coupled with an effective therapeutic level of gene-modified HSCs [28]. Without new answers to these current challenges, the hope of safe HSC gene therapy will be severely limited.

## Epigenetics and HSCs

Epigenetic modifications play a key role in regulating cell self-renewal and differentiation as well as tissue development [29]. There are three main categories of modifications that can induce gene expression regulation which include DNA methylation, histone modifications, and nucleosome positioning [29]. New studies have shown that epigenetic mechanisms, involving the regulation of both gene expression and DNA recombination, usually through the control of chromatin assembly, contribute to establishing HSC unique properties [29-31]. To date, the mechanisms involved in controlling the manner in which genes are silenced or made accessible for transcription in the hematopoietic system are still largely unknown.

The most widely studied epigenetic modification in humans is cytosine methylation [29]. The majority of the time, DNA methylation occurs at regions called CpG islands, or clusters of CpG dinucleotides of 0.5-2 kilobases [29]. These sequences are usually constitutively non-methylated in all animal cell types. The DNA methyltransferases (DNMTs) are the enzymes responsible for regulating the initiation and maintenance of the methyl marks [29]. DNA methylation is a strong inhibitor of gene expression which has a profound effect on HSC characteristics. Histones and nucleosomes also play a key role in regulating gene expression. Histone post-transcriptional modifications occur predominantly in histone tails. These modifications include phosphorylation, acetylation, methylation, ubiquitination, ADP-ribosylation, and SUMOylation [29-31]. Nucleosomes serve as barriers to transcription by blocking access of transcription factors and activators to their sites on DNA [29].

Recent studies have shown that the function of a robust stimulator of HSCs/HPCs, SALL4, is associated with epigenetic machinery [1,30]. SALL4 may provide specific targets for histone deacetylase enzymes and DNA methyltransferases resulting in the generation of specific histone deacetylation or DNA methylation patterns associated with HSC/HPC expansion [30].

Polycomb group (PcG) and Trithorax group (TrxG) proteins have emerged as significant epigenetic regulators. These groups act antagonistically to either promote or repress transcription through regulation of specific amino acid modifications in histones [29]. Polycomb group proteins function in two main complexes in vertebrates known as Polycomb Repressive Complex (PRC) 1 and PRC2 [31]. BMI1, a component of PRC1, has been found to be a key factor in cell self-renewal [31]. BMI1 is expressed in HSCs and is retained within lymphoid cells, but its expression decreases upon differentiation towards myeloid or erythroid lineages [31]. Studies have shown that overexpression of BMI1 in cord blood CD34+ cells resulted in stem cell maintenance[32]. Finally, after a culture period of 10 days, BMI1-overexpressing cells display improved engraftment in NOD-SCID mice [32].

## Future promise of expansion of HSCs

While there are numerous clinical trials and assays testing the efficacy and safety for a number of molecules ability to expand HSCs, an ideal factor to increase the number of HSCs in vitro without limiting their in vivo regeneration capabilities has yet to be found. The main difficulties surrounding HSC expansion therapy include long-term engraftability and the translation of in vitro observations into successful in vivo outcomes. However, our increasing knowledge base of the molecular mechanisms underlying the function of HSCs within humans should shed light upon what strategies may lead to the development of an efficient gene therapy via HSCs. Advancements in the methods used to expand HSCs should greatly improve disease outcomes in the future. Further developments in the field coupled with gene therapy and stem cell transplant should encourage progression in cord blood transplantation, therapy for hematologic diseases, and issues of efficacy and safety.

## Competing interests

The authors declare that they have no competing interests.

## Authors’ contributions

JAS, MRS, GM: Manuscript writing. WL,YM: Discussion and manuscript writing. RL: Discussion. JRA: Directed and conceived study, discussion, and manuscript writing. All authors read and approved the final manuscript.
